# Somatic alterations of targetable oncogenes are frequently observed in *BRCA1/2* mutation negative male breast cancers

**DOI:** 10.18632/oncotarget.12272

**Published:** 2016-09-27

**Authors:** Piera Rizzolo, Anna Sara Navazio, Valentina Silvestri, Virginia Valentini, Veronica Zelli, Ines Zanna, Giovanna Masala, Simonetta Bianchi, Marco Scarnò, Stefania Tommasi, Domenico Palli, Laura Ottini

**Affiliations:** ^1^ Department of Molecular Medicine, Sapienza University of Rome, Rome, Italy; ^2^ Molecular and Nutritional Epidemiology Unit, Cancer Research and Prevention Institute (ISPO), Florence, Italy; ^3^ Division of Pathological Anatomy, Department of Medical and Surgical Critical Care, University of Florence, Florence, Italy; ^4^ CINECA (Inter University Consortium for Super Computing), Rome, Italy; ^5^ Molecular Genetics Laboratory, Istituto Tumori “Giovanni Paolo II”, Bari, Italy

**Keywords:** male breast cancer, somatic mutations, gene copy number variation, germ-line *BRCA1/2* mutations, oncogenes

## Abstract

Male breast cancer (MBC) is a rare disease. Due to its rarity, MBC research and clinical approach are mostly based upon data derived from its largely known female counterpart. We aimed at investigating whether MBC cases harbor somatic alterations of genes known as prognostic biomarkers and molecular therapeutic targets in female breast cancer.

We examined 103 MBC cases, all characterized for germ-line *BRCA1/2* mutations, for somatic alterations in *PIK3CA*, *EGFR*, *ESR1* and *CCND1* genes.

Pathogenic mutations of *PIK3CA* were detected in 2% of MBCs. No pathogenic mutations were identified in *ESR1* and *EGFR*. Gene copy number variations (CNVs) analysis showed amplification of *PIK3CA* in 8.1%, *EGFR* in 6.8% and *CCND1* in 16% of MBCs, whereas deletion of *ESR1* was detected in 15% of MBCs. Somatic mutations and gene amplification were found only in *BRCA1/2* mutation negative MBCs.

Significant associations emerged between *EGFR* amplification and large tumor size (T4), ER-negative and HER2-positive status, between *CCND1* amplification and HER2-positive and MIB1-positive status, and between *ESR1* deletion and ER-negative status.

Our results show that amplification of targetable oncogenes is frequent in *BRCA1/2* mutation negative MBCs and may identify MBC subsets characterized by aggressive phenotype that may benefit from potential targeted therapeutic approaches.

## INTRODUCTION

Male breast cancer (MBC) is a rare disease, representing less than 1% of all breast cancers (BCs) and less than 1% of all cancers in men [1]. Germ-line mutations in the high-penetrance BC genes, *BRCA1* and, mainly, *BRCA2* play a major role in MBC susceptibility and about 10% of MBCs are associated with mutations in these genes [2].

Due to its rarity, MBC research and patient management are mostly based upon data derived from its largely known female counterpart. To date treatment strategies for MBC patients generally follow those for female patients. As most breast cancers in men are hormone receptor-positive, current therapeutic options mainly include hormone therapy that is usually recommended for MBC patients following the same recommendations as hormone-dependent-BC in women [3].

MBC shares some similarities with post-menopausal estrogen receptor (ER)-positive female breast cancer (FBC), however increasing evidence indicates that, on clinical and molecular level, MBC may be a heterogeneous disease, different from FBC [4–6]. Compared to FBC, MBC occurs later in life, with higher stage, lower grade and more estrogen/progesterone receptor (ER/PR) positivity [7–8].

To date, the majority of MBC studies focused on germ-line mutational analysis and gene expression profiling, while there are only few studies investigating somatic alterations in MBCs [4–5, 9]. By contrast, accumulating data have provided a landscape of somatic alterations with possible clinical relevance and several somatic mutations with potential prognostic and therapeutic significance are known in FBC [10–13]. In addition to gene mutations, gene copy number variations (CNVs) have been reported as an important mechanism in the development and progression of cancer and could serve as potential prognostic biomarkers and molecular therapeutic targets [14–18].

A comprehensive portrait of the genetic landscape of FBCs has demonstrated that Phosphatidylinositol 3-kinase gene (*PIK3CA*) is the most commonly mutated gene and two mutation hotspot regions in exons 9 and 20 have been identified [19–20]. Mutations of *PIK3CA* have been suggested to have prognostic value and to confer resistance to hormone therapy and to downstream mTOR inhibitors [11, 21]. The proportion of FBCs exhibiting *PIK3CA* mutations ranges between 20-25%, and, notably, mutations occur approximately in 40% of ER-positive FBCs [11, 19–23]. To date four studies investigated *PIK3CA* mutations in MBC [6, 24–26]. Overall *PIK3CA* mutation frequency observed in MBC was lower than FBC. In addition to somatic mutations, gene amplification of *PIK3CA* is reported in about 10% of FBC [27–29]. Among targetable genes, the epidermal growth factor receptor (*EGFR*) is one of the first important molecular targets identified in BC [10, 30–31]. *EGFR* gene CNVs in FBC range from 8 to 10%, [32–33] while the proportion of FBCs exhibiting *EGFR* somatic mutations ranges from 2 to 15% [34–36]. Notably a higher rate of activating *EGFR* mutations is detected in *BRCA1/2* mutation positive tumors, supporting the hypothesis that carcinogenic processes may be dependent on the germ-line phenotype [34]. *CCND1* is an estrogen-responsive gene that enhances ER-mediated gene transcription. The product of *CCND1*, cyclin D1, provide growth advantage to cancer cells and contribute toward resistance to endocrine therapy in ER-positive cancers. In FBC, *CCND1* is amplified in 5–20% of primary BCs, typically ER-positive BC, and is a prognostic biomarker with a potential therapeutic role [12, 13, 15, 37, 38]. As MBC is an estrogen-driven disease and is most frequently ER-positive, the investigation of *CCND1* alterations might be relevant in male breast tumors [8]. Recently, *CCND1* amplification has been shown to be an independent prognostic factor in MBC [39]. Somatic mutations of *CCND1* are not a frequent alteration in cancer, and, in particular have not been reported in BC [19, 40].

Interestingly, crosstalk between ER and PI3K pathway may increase estrogen-induced and ligand-independent ER transcriptional activity [41]. Recurrent activating mutations of *ESR1*, the gene encoding the estrogen receptor alpha (ERα), have been identified in hormone therapy-refractory ER-positive metastatic FBC [42–44]. CNVs of *ESR1* have been largely investigated in FBC, although with controversial results [45]. Data emerging from studies evaluating *ESR1* somatic alterations in MBC suggest that *ESR1* alterations may be gender specific [39].

Although knowledge on somatic landscape of MBC is increasing, comprehension on the role of somatic alterations of specific genes with potential prognostic and therapeutic significance need to be further investigated in MBCs characterized for *BRCA1/2* mutations. In this study, we focused on somatic mutations and CNVs of *PIK3CA*, *EGFR*, *ESR1* and *CCND1* genes by examining a large series of MBCs screened for *BRCA1/2* germ-line mutations.

## RESULTS

### Mutational screening

**Figure 1:**
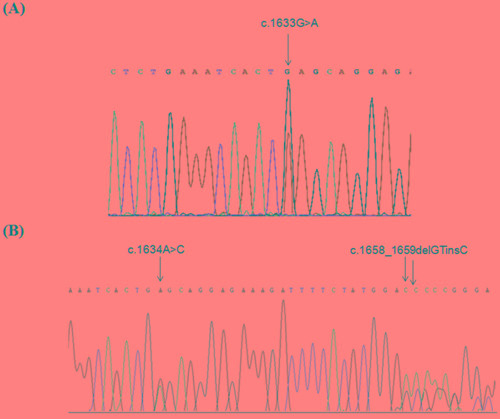
*PIK3CA* mutations identified in MBCs Partial electropherograms from direct sequencing of *PIK3CA* exon 9 in a case harboring the c.1633G>A (p.E545K) mutation **A.** and in a case harboring c.1634A>C (p.E545A) and 1658delGTinsC (p.S553Tfs*7) mutations **B.**

Mutational screening of *PIK3CA* was performed in 102 primary breast cancers and one lymph-node metastasis. Two mutations, both located in exon 9, were detected: the c.1633G>A (p.E545K) was identified in two MBC cases and the c.1634A>C (p.E545A) in two additional cases (Table 1). In these latter cases, a second mutation, c.1658_1659delGTinsC (p.S553Tfs*7), also located in exon 9, was detected (Figure 1).

**Table 1:** Summary of results from *PIK3CA*, *EGFR* and *ESR1* mutational analysis

Gene	Exon	Nucleotide change	Amino acid change	Sample
PIK3CA	9	c.1633G>A	p.E545K^a^	primary tumor
PIK3CA	9	c.1634A>C; c.1658_1659delGTinsC	p.E545A^a^; p.S553Tfs*7^a^	primary tumor
PIK3CA	20	c.3140A>G	p.H1047R^a^	metastatic lymph node
EGFR	20	c.2361G>A	p.Q787Q^b^	primary tumor
EGFR	23	c.2748C>T	p.N916N^b^	primary tumor
ESR1	4	c.975G>C	p.P325P^b^	primary tumor

a pathogenic mutation;

b synonymous variant.

All mutations and variants identified are recorded on COSMIC database (http://cancer.sanger.ac.uk/cosmic).

**Figure 2:**
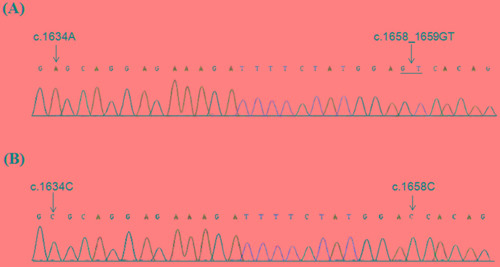
Specific sequencing of *PIK3CA* exon 9 and pseudogene Partial electropherograms from direct sequencing of DNA region using primers specific for *PIK3CA* exon 9 **A.** and for pseudogene **B.**

To exclude that *PIK3CA* pseudogene, retaining high homology with the sequence of exon 9 (https://blast.ncbi.nlm.nih.gov/Blast.cgi?PAGE_TYPE=BlastSearch), might affect *PIK3CA* mutation screening, we performed amplification and sequencing analysis using primers specific for the *PIK3CA* gene and pseudogene (Supplementary Table S1). This analysis showed that both c.1634G>A and c.1658_1659delGTinsC were due to the pseudogene amplification (Figure 2).

Overall, *PIK3CA* mutations were identified in 2 out of 102 (2%) primary MBCs examined. Both these MBCs were from *BRCA1/2* mutation negative patients with no family history of BC, were invasive ductal carcinomas, and presented with high grade (G3), ER-positive, HER2-negative and lymph node positive status.

An additional *PIK3CA* mutation located in exon 20, the c.3140A>G (p.H1047R), was detected in the metastatic lymph node sample examined from a MBC case whose primary tumor was *PIK3CA* mutation negative (Table 1).

Mutational analysis of *EGFR* identified two synonymous variants (Table 1): the c.2361G>A (p.Q787Q) occurring in 15 of 102 cases (14.7%), and c.2748C>T (p.N916N) occurring in one case. Mutational analysis of *ESR1* identified one synonymous variant c.975G>C (p.P325P) in 15/70 cases (21.4%). No pathogenic mutations were identified in *ESR1* and *EGFR* genes (Table 1).

### Gene copy number variation analysis

Overall, gene CNV analysis showed amplification of *PIK3CA*, *EGFR* and *CCND1*, and deletion of *ESR1.* Specifically, amplification of *PIK3CA* was detected in 6/74 (8.1%), *EGFR* in 5/74 (6.8%) and *CCND1* in 10/62 (16%) of the MBC cases analyzed. Overall, gene amplifications were detected in MBCs from *BRCA1/2* mutation negative patients. Deletion of *ESR1* was shown in 11/73 (15%) of the cases, including one case with *BRCA2* germ-line mutation. Co-amplification of *PIK3CA*, *EGFR* and *CCND1* was observed in two MBC cases and co-amplification of *EGFR* and *CCND1* in one case. Furthermore, one case showed both *PIK3CA* mutation and *CCND1* amplification.

**Figure 3:**
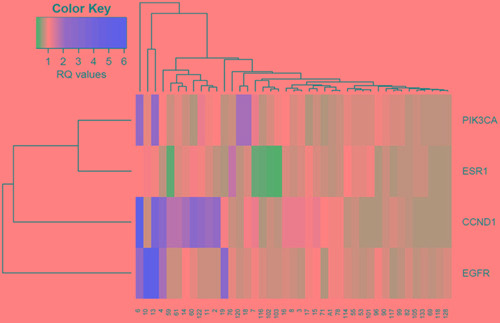
Unsupervised hierarchical clustering analysis of CNVs in *EGFR, PIK3CA, ESR1* and *CCND1* genes in male breast tumors

The overall CNV profile was investigated by unsupervised hierarchical clustering analysis performed on 41 MBC cases, for which data on CNVs of all genes examined were available. Hierarchical cluster analysis revealed two groups of cases (Figure 3). One group characterized by no CNV or CNV in only one gene, mainly *CCND1* amplification, and a second group characterized by CNVs in multiple genes. Notably, this second group included cases characterized by aggressive pathologic features, including high tumor grade (G3), large tumor size (T4) and lymph node involvement.

### Associations between somatic alterations and clinical-pathologic features

As shown in Table 2, statistically significant differences emerged in the distribution of *EGFR*, *CCND1* and *ESR1* CNVs according to ER, HER2, MIB1 and tumor size (T) status. In particular, CNVs of *EGFR* were associated to ER, HER2, MIB1 and T status, of *CCND1* to HER2 and MIB1 status and of *ESR1* to ER status.

**Table 2:** Frequencies of clinical-pathologic features considered in 103 primary male breast tumors crossed with gene copy number variations (CNVs)

Parameter	Total cases (%)	Gene CNVs							
		*PIK3CA*		*EGFR*		*CCND1*		*ESR1*	
		amp/N^a^	p^b^	amp/N^a^	p^b^	amp/N^a^	p^b^	del/N^a^	p^b^
**FH^c^ of breast/ovarian cancer**									
Negative	75 (72.8)	6/53	0.108	5/56	0.189	6/49	0.106	7/52	0.546
Positive	28 (27.2)	0/21		0/18		4/13		4/21	
**Personal history of cancer**									
Negative	77 (74.8)	5/56	0.648	5/56	0.189	8/46	0.647	9/50	0.302
Positive	26 (25.2)	1/18		0/18		2/16		2/23	
***BRCA1/2 status***									
*BRCA1* mutated	2 (2)	0/1	0.673	0/1	0.789	0/1	0.528	0/1	0.872
*BRCA2* mutated	9 (8.7)	0/7		0/5		0/5		1/5	
*BRCA1/2* wild-type	92 (89.3)	6/66		5/68		10/56		10/67	
**Hystological type**									
Infiltrating ductal carcinoma	82 (83.7)	5/59	0.277	5/60	0.352	10/52	0.15	9/64	0.702
Other	16 (6.3)	0/13		0/10		0/9		1/10	
**ER**									
Negative	15 (14.8)	2/11	0.192	3/10	**0.002**	2/10	0.736	4/9	**0.009**
Positive	86 (85.2)	4/62		2/62		8/55		7/64	
**PR**									
Negative	24 (23.8)	1/20	0.538	3/18	0.061	5/42	0.159	3/16	0.641
Positive	77 (76.2)	5/53		2/54		5/19		8/57	
**HER2**									
Negative	69 (74.2)	2/46	0.051	1/46	**0.012**	4/41	**0.019**	10/53	0.201
Positive	24 (25.8)	4/21		4/20		6/17		1/17	
**MIB1 (Ki67)**									
Negative	60 (61.2)	1/38	0.064	0/38	**0.013**	2/30	**0.032**	8/44	0.293
Positive	38(38.8)	5/34		5/33		8/29		3/30	
**Hystological grade**									
G1+G2	61 (64.2)	4/43	0.818	2/45	0.199	4/37	0.135	8/47	0.383
G3	34 (35.8)	2/26		3/23		5/19		2/22	
**Lymph nodes**									
Not involved	43 (55.8)	1/29	0.249	2/34	0.487	3/24	0.517	3/31	0.513
Involved	34 (44.2)	3/26		3/28		5/26		4/26	
**Tumor size**									
T1-T2	67 (83.6)	3/49	0.151	2/56	**0.006**	6/44	0.21	9/51	0.132
T4	13 (16.4)	2/10		3/11		3/10		0/11	

a amp: amplified; del: deleted; N: number of tumors that were successfully analyzed;

b p value from Chi-square test;

c FH: Family history of breast and/or ovarian cancer.

In bold p-value <0.05, considered statistically significant.

By linear logistic regression analysis a statistically significant association was confirmed between *EGFR* amplification and ER-negative status (p=0.01), HER2-positive status (p=0.03) and T4 (p=0.01). By using a stepwise logistic regression, in which ER, HER2, MIB1 and T status were included, the dependency of these four characteristics in relation to *EGFR* amplification was analyzed, and only ER was selected as a significant effect (p=0.044). Furthermore, by linear logistic regression analysis significant association was confirmed between *CCND1* amplification and HER2-positive (p=0.0005) and MIB1-positive (p=0.04) status, and between *ESR1* deletion and ER-negative status (p=0.01).

## DISCUSSION

In this study we investigated somatic mutations and CNVs of *PIK3CA, EGFR, CCND1* and *ESR1* in a large series of MBCs characterized for germ-line *BRCA1* and *BRCA2* mutations. We found that gene CNVs are frequent in *BRCA1/2* mutation negative MBCs and allow for the identification of MBC subsets characterized by pathologic characteristics suggestive of aggressive phenotype. By contrast, somatic mutations were rarely detected and were identified only in *PIK3CA* gene. We did not detect somatic mutations in *ESR1* and *EGFR*, indicating that *EGFR* and *ESR1* mutations are infrequent in MBC and confirming recent data showing no deleterious somatic mutations of these genes in MBC [6].

In our series *PIK3CA* mutations were identified in 2% of MBCs. Our data indicate a lower *PIK3CA* mutation frequency compared with previous data on MBC. *PIK3CA* mutations have been reported in 18-20% of MBCs, not characterized for *BRCA1/2* mutation status, and in about 10% of familial *BRCA1/2* mutation negative MBCs [6, 24–26]. Though differences in *PIK3CA* mutation frequency may be due to different screening methods and sample selection criteria, it is worth noting that our results refer to a MBC series that represents the twice of those previously reported and included hereditary, familial and sporadic cases, all characterized for *BRCA1/2* mutation status. Overall, in our series *PIK3CA* mutations were identified in *BRCA1/2* mutation negative MBCs, thus confirming the hypothesis that *PIK3CA* mutation status is dependent on germ-line genotypes of MBC [25–26].

We detected the co-existence of two *PIK3CA* mutations, c.1634G>A (p.E545A) and c.1658_1659delGTinsC (p.S553Tfs*7) both located in exon 9, in two independent MBC cases. The concurrence of these two somatic mutations has been previously reported in large B cell lymphoma and non-small cell lung cancer [46–47]. Moreover, the concurrence of these mutations as germ-line mutations has been suggested to predispose to Cowden and Cowden like syndromes [48]. Double mutations in *PIK3CA* are rare phenomena [49]. To exclude that a pseudogene of *PIK3CA* located on chromosome 22q11.2 and that retains about 95% homology with the sequence of *PIK3CA* exons 9-13 (https://blast.ncbi.nlm.nih.gov/Blast.cgi?PAGE_TYPE=BlastSearch), might have influenced the accuracy of mutation detection, we performed true-specific *PIK3CA* and pseudogene sequencing analysis. These analyses showed that both c.1634A>C (p.E545A) and c.1658_1659delGTinsC (p.S553Tfs*7) were due to the pseudogene amplification. Similar observations have been reported in previous studies [50–52]. Therefore, when interpreting data corresponding to *PIK3CA* mutations, attention should be given to the possible co-amplification of *PIK3CA* gene and its pseudogene.

Interestingly, we detected *PIK3CA* c.3140A>G (p.H1047R) hotspot activating mutation in a metastatic lymph node of a MBC case that did not harbor *PIK3CA* mutation in the corresponding primary breast tumor. A different *PIK3CA* mutation status between primary tumors and corresponding metastatic lesions has been previously reported in FBC [53–54], indicating that *PIK3CA* mutations may be acquired during tumor progression. This finding may be relevant for clinical management, suggesting the need of assessing *PIK3CA* mutation status in metastatic lesions for the selection of *PIK3CA* inhibitor therapy.

We reported the occurrence of *PIK3CA* amplification at higher frequency than that previously reported in MBC [26]. The frequency of *PIK3CA* amplification found in our study is consistent with that reported in FBC [27–29]. As for *PIK3CA* mutations, *PIK3CA* amplification was found in *BRCA1/2* mutation negative MBCs.

In our MBC series *EGFR* amplification was found at a higher frequency compared to previous data on MBC [26, 39] and FBC [32–33]. Furthermore, we showed that *EGFR* amplification was associated with ER-negative status, HER2-positive status and large tumor size (T4). These findings suggest that *EGFR* amplification could represent a prognostic marker in MBC and could identify a subset of MBC patients at high risk for adverse prognosis. Our data also suggest that this subset of MBCs (i.e. ER-negative and HER2-positive) might benefit from target therapy. Interestingly, there is evidence showing that therapies combining HER2 inhibitors with EGFR inhibitors, such as trastuzumab with lapatinib, may produce promising results in MBC treatment [55]. Overall, the association between *EGFR* amplification and ER-negative status suggest that EGFR signaling pathway may play a role in male breast carcinogenesis, mainly in estrogen-insensitive breast tumors. Interestingly, association between *EGFR* amplification and ER-negative status was also reported in FBC [32].

*CCND1* amplification was the most frequent CNV observed in our MBC series, thus supporting the hypothesis that this gene may play a relevant role in male breast carcinogenesis, particularly in *BRCA1/2* mutation negative cases. Our results are in agreement with previous data showing that *CCND1* was one of the genes that most frequently showed copy number gain in MBC [39]. In our series, *CCND1* amplification identified a subset of MBCs characterized by HER2-positive and MIB1-positive status, pathologic features suggestive of aggressive phenotype. These findings are in line with the function of *CCND1* protein product and are consistent with previous data showing that MBCs with *CCND1* copy number gain tended to have a higher mean mitotic count compared to tumors without *CCND1* amplification [39]. Notably, *CCND1* amplification seems to be an independent prognostic factor in MBC correlating with poor survival [39]. Amplification of *CCND1* has also been associated with poor prognosis in ER-positive FBC [12, 37]. As *CCND1* is an estrogen-responsive gene, one possible mechanism by which its amplification could lead to a poor prognosis is by conferring resistance to hormone therapy [13, 56]. These findings may acquire great importance in MBC, for which endocrine therapy is the most common treatment [3].

*ESR1* deletion was reported at high frequency in our MBC series. Our results are consistent with data reporting gene deletion in MBC [39] and confirm that *ESR1* amplification is not a characteristic alteration of MBC. By contrast, *ESR1* amplification has been reported, although with controversial results, in FBC in which *ESR1* deletion does not appear to be a significant event [45]. In our series, a significant association emerged between *ESR1* gene deletion and ER-negative status. Interestingly several data suggest that low-level of *ESR1* expression may be one of the mechanisms responsible for tamoxifen resistance in ER-positive FBC [57]. These observations confirm the utility of our results in the identification of MBC subsets that may need specific therapeutic approach.

In agreement with the hypothesis that tumors rising on gene amplification are more likely to develop multiple amplifications as they have greater genetic instability [15], in this study we identified co-amplification of genes analyzed. By cluster analysis, we identified a group of cases characterized by co-amplification of two or more genes, suggesting the need to develop new therapeutic strategies involving multiple targets.

Overall, our data indicate that genes known to be important prognostic biomarkers and molecular therapeutic targets in FBC are also important in MBC. For instance, adjuvant trastuzumab, a monoclonal antibody that selectively targeted HER2 and is used as therapeutic agent in HER2-positive FBCs, can be considered for high-risk HER2-positive MBCs [58]. However, differences emerged between male and female breast cancers, emphasizing the importance of identifying biomarkers based on research in MBC.

In conclusion our results showed that CNVs of *PIK3CA*, *EGFR*, *CCND1* and *ESR1* genes may play a relevant role in male breast carcinogenesis and could lead to the identification of specific molecular profiles. In particular, amplification of targetable oncogenes is frequent in *BRCA1/2* mutation negative MBCs and may identify biomarkers relevant for clinical management and targeted therapeutic approach.

## MATERIALS AND METHODS

### Patients and genomic DNA

A series of 103 primary male breast tumors was included in the study. A metastatic lymph node sample was available for one case and was also included in the analyses. All MBC cases were characterized for germ-line *BRCA1* and *BRCA2* mutations and for the main clinical-pathologic features, including: age at diagnosis, family and personal history of cancer, tumor histological type, grade (G), nodal status, tumor size (T), estrogen receptor (ER), progesterone receptor (PR), MIB1 (Ki67) and HER2 expression (Table 2). The expression of ER, PR, and Ki67/MIB1 was scored based on the percentages of positive nuclei (ER/PR positive if >10%; Ki67/MIB1 high if >20%) over the total number of counted cancer cell nuclei HER2 positivity was defined as a score of 3+ using immunohistochemistry (IHC) test, or amplification shown by fluorescence in situ hybridization (FISH), in equivocal cases [59–60].

Genomic DNA was isolated from microdissected formalin fixed paraffin-embedded (FFPE) and fresh frozen tumor sections. DNA from FFPE sections was extracted using DNA/RNA FFPE kit (Qiagen Inc., Venlo, Netherlands) according to the manufacturer's instructions. DNA from fresh frozen samples was extracted by following standard phenol-chloroform extraction protocol. For some cases the amount of DNA was inadequate to carry out all molecular analyses.

The participants signed an informed consent form with a detailed description of the study protocol. The study was approved by The Local Ethical Committee (Sapienza University of Rome, Protocol 264/12).

### Mutational screening

Mutational analysis of *PIK3CA*, *EGFR* and *ESR1* was performed mainly by direct sequencing and by SSCP (Single Strand Conformation Polymorphism). Genomic DNA from each tumor sample was amplified using primers covering exons in which is reported to cluster the great majority of mutations (Supplementary Table S1), in particular exons 9 and 20, coding the helical and kinase domains of *PIK3CA*, respectively [61]; exons from 18 to 24, coding kinase domain of *EGFR*; exons from 4 to 8, coding the ligand binding domain, of *ESR1*.

To overcome the interference of a pseudogene of *PIK3CA*, we also designed specific primers for both *PIK3CA* gene and pseudogene (Supplementary Table S1). DNA samples from cell lines (HCC15 and HCT16) with known mutations in exons 9 (c.1633G>A, p.E545K) and 20 (HCT-116 c.3140 A>G, p.H1047R) of *PIK3CA*, were used as positive control.

### Gene copy number variation analysis

*PIK3CA, EGFR, ESR1* and *CCND1* gene copy number variations (CNVs) were analyzed by Real-Time PCR, using TaqMan probe-based technology (Applied Biosystems, Foster City California, USA). The RNaseP as housekeeping gene and a normal male breast tissue sample as calibrator were used. TaqMan Copy number Assays (Hs02708380_cn for *PIK3CA*, Hs00756893_cn for *EGFR*, Hs02577150_cn for *ESR1* and Hs01818912_cn for *CCND1*) and TaqMan Copy number Reference Assay human RNaseP, containing primers and probe mix for target and housekeeping genes respectively, were used (Applied Biosystems, Foster City California, USA). The analysis of relative gene expression data was performed, using the 2^−ΔΔCt^ method. The fold change in studied gene copy number, normalized to endogenous control, was calculated using RQ=2^−ΔΔCt^ [62–63]. Values of RQ ≥2 were considered amplified and values of RQ <0.5 were considered deleted.

### Statistical analysis

Unsupervised hierarchical clustering was performed to analyze relevant clusters characterized by co-amplification/deletion in *EGFR, PIK3CA, ESR1* and *CCND1.*

Chi square test and logistic regression models were performed in order to evaluate the potential associations between each gene alteration (mutations and CNVs) and clinical-pathologic features. A P value ≤0.05 was considered statistically significant. All statistical analyses were performed with the R software (www.r-project.org).

## SUPPLEMENTARY MATERIALS TABLE




