# Supplementary material for: Somatic alterations of targetable oncogenes are frequently observed in BRCA1/2 mutation negative male breast cancers
Source: Oncotarget. 2016 Sep 27;7(45):74097–106. doi: 10.18632/oncotarget.12272 (PMC5342038; doi:10.18632/oncotarget.12272)
Supplement: Supplementary file 1 [file oncotarget-07-74097-s001.pdf]

Somatic alterations of targetable oncogenes are frequently observed in *BRCA1/2* mutation negative male breast cancers

SUPPLEMENTARY TABLE

Supplementary Table S1: List of primer sets used for *PIK3CA*, *EGFR*, *ESR1* and *PIK3CA-PSEUDOGENE* PCR-amplification and Sanger Sequencing

See Supplementary File 1
