# Supplementary material for: Somatic alterations of targetable oncogenes are frequently observed in BRCA1/2 mutation negative male breast cancers
Source: Oncotarget. 2016 Sep 27;7(45):74097–106. doi: 10.18632/oncotarget.12272 (PMC5342038; doi:10.18632/oncotarget.12272)
Supplement: Supplementary file 2 [file oncotarget-07-74097-s002.docx]

**Table S1: List of primer sets used for *PIK3CA*, *EGFR*, *ESR1* and *PIK3CA-PSEUDOGENE* PCR-amplification and Sanger Sequencing**

| ***PIK3CA*** | Sequence ('5→3') |
| --- | --- |
| Exon 9-Fw | GATTGGTTCTTTCCTGTCTCTG |
| Exon 9-Rv | CCACAAATATCAATTTACAACCATTG |
| Exon 20a-Fw | TGGGGTAAAGGGAATCAAAAG |
| Exon 20a-Rv | CCTATGCAATCGGTCTTTGC |
| Exon 20b-Fw | TTGCATACATTCGAAAGACC |
| Exon 20b-Rv | GGGGATTTTTGTTTTGTTTTG |
| ***EGFR*** |  |
| Exon18- Fw | AGGGCTGAGGTGACCCTTGT |
| Exon18-Rv | TCCCCACCAGACCATGAGAGG |
| Exon 19- Fw | ACCATCTCACAATTGCCAGTTAAC |
| Exon 19-Rv | GAGGTTCAGAGCCATGGACC |
| Exon 20- Fw | GATCGCATTCATGCGTCTTCACC |
| Exon 20-Rv | TTGCTATCCCAGGAGCGCAGACC |
| Exon 21- Fw | TCACAGCAGGTCTTCTCTGTTT |
| Exon 21-Rv | ATGCTGGCTGACCTAAAGCC |
| Exon 22- Fw | AATTAGGTCCAGAGTGAGTTAAC |
| Exon 22-Rv | ACTTGCATGTCAGAGGATATAATG |
| Exon 23-Fw | CATCAAGAAACAGTAACCAGTAATG |
| Exon 23-Rv | AAGGCCTCAGCTGTTTGGCTAAG |
| Exon 24- Fw | TTGACTGGAAGTGTCGCATCACC |
| Exon 24-Rv | CATGTGACAGAACACAGTGACATG |
| ***ESR1*** |  |
| Exon 4a- Fw | TTTTTTCCACCTGTGTTTTCAG |
| Exon 4a-Rv | AAGGTTGGCAGCTCTCATGTCT |
| Exon 4b- Fw | GAAACACAAGCGCCAGAGAG |
| Exon 4b-Rv | GGGCTCAGCATCCAACAA |
| Exon 4c- Fw | CCGCTCATGATCAAACGCTCTA |
| Exon 4c-Rv | TCTGCCAGGTTGGTCAGTAAGC |
| Exon 4d- Fw | TGCTGAGCCCCCCATACTCTATT |
| Exon 4d-Rv | TTAAAAGCTGCGCTTCGCATT |
| Exon 5a- Fw | TTGAGTCAGCAGGGTTTTTCTT |
| Exon 5a-Rv | AAGAGCAAGTTAGGAGCAAACAGT |
| Exon 5b- Fw | TGTGGATTTGACCCTCCATGAT |
| Exon 5b-Rv | ACTCCTAAGCTACAGCCAGGTCAC |
| Exon 6a- Fw | CATGTCTTGTGGAAGATTTTCTGT |
| Exon 6a-Rv | AAACTCCTCTCCCTGCAGATT |
| Exon 6b- Fw | TGCTGCTGGCTACATCATCTC |
| Exon 6b-Rv | CATCCAGCATTGAGTTATCTTGTG |
| Exon 7a- Fw | CCTCTTTGAGCTTCTCTCTCTCAC |
| Exon 7a-Rv | TGCCTTGGCCATCAGGTG |
| Exon 7b- Fw | CGAGTCCTGGACAAGATCACA |
| Exon 7b-Rv | GTAGGAAGCCCACAGATGCC |
| Exon 8a- Fw | TCGGGTTGGCTCTAAAGTAGTCC |
| Exon 8a-Rv | TAGTGGGCGCATGTAGGC |
| Exon 8b- Fw | CAAGAACGTGGTGCCCCTCTAT |
| Exon 8b-Rv | GCAAGGAATGCGATGAAGTAGAG |
| Exon 8c- Fw | CTACATGCGCCCACTAGCC |
| Exon 8c-Rv | GCCTCCCCCGTGATGTAAT |
| Exon 8d- Fw | AAGCCACTTGGCCACTGC |
| Exon 8d-Rv | GCAGCAGGGATTATCTGAACC |
| ***PIK3CA exon9-specific*** |  |
| Forward | ATTTCTACACGAGATCCTCTCTCT |
| Reverse | CCATTTTAGCACTTACCTGTGAC |
| ***Pseudogene-specific*** |  |
| Forward | ATTTCTACACGAGATCCTCTCTCT |
| Reverse | CCATTTTAGCACTTACCTGTGGT |

Primers used for *PIK3CA* were previously reported by *Kang et al PNAS 2005*.

*EGFR* and *ESR1* primers were designed using Primer3Plus (www.bioinformatics.nl/primer3plus).

Primers used for *PIK3CA* exon 9-specific and *PSEUDOGENE*-specific amplification were previously reported by *Baker et al Journal of Molecular Diagnostics 2012.*
